# Normotensive hypokalemic primary hyperaldosteronism mimicking clinical features of anorexia nervosa in a young patient: A case report

**DOI:** 10.1097/MD.0000000000020826

**Published:** 2020-07-17

**Authors:** Yen-Chu Huang, Ming-Hsien Tsai, Yu-Wei Fang, Mei-Lan Tu

**Affiliations:** a Division of General Medicine, Department of Internal Medicine; b Division of Nephrology, Department of Internal Medicine, Shin-Kong Wu Ho-Su Memorial Hospital; c Fu-Jen Catholic University School of Medicine, Taipei, Taiwan (ROC).

**Keywords:** anorexia nervosa, hypokalemia, normal blood pressure, primary hyperaldosteronism

## Abstract

**Rationale:**

The typical clinical presentations of patients with primary aldosteronism (PA) include generalized weakness, fatigue, high blood pressure, and potassium deficiency. However, normotensive PA is rare. Therefore, an atypical presentation of normal blood pressure is a challenge for the diagnosis and treatment of PA.

**Patient concerns:**

A 43-year-old, thin, and tall woman (body mass index, 18.6 kg/m^2^) with generalized weakness for 1 day presented to our emergency department, where hypokalemia was a significant finding. The initial diagnosis was anorexia nervosa with the evidence of renal potassium wasting with low urinary sodium and chloride levels, metabolic alkalosis, normal blood pressure, and low body mass index. However, neither vomiting features nor other specific induced vomiting features were noted.

**Diagnoses:**

The laboratory examination revealed high plasma aldosterone level, low plasma renin activity, and extremely high aldosterone-to-renin ratio indicating the diagnosis of PA, confirmed via adrenal computed tomography.

**Interventions:**

Surgical adrenalectomy was performed. Pathological diagnosis was a benign cortical adenoma.

**Outcomes:**

Patient's serum potassium level and hormonal status became normalized after surgical removal of adrenal adenoma. She fully recovered without any further sequelae.

**Lessons:**

It is too early to rule out PA based on the presence of normal blood pressure in a patient with metabolic alkalosis and renal wasting hypokalemia. Moreover, PA should be considered in a normotensive patient with an unknown hypokalemic etiology to avoid delayed diagnosis and treatment.

## 1 Introduction

The most common cause of secondary hypertension is primary aldosteronism (PA), caused by increased aldosterone secreted in the zona glomerulosa of the adrenal cortex.^[1–3]^ The typical form of PA is characterized by hypertension and hypokalemia. Although some patients with PA may not have hypokalemia, the vast majority of PA patients suffer from hypertension. Normotensive PA is rare.^[1–4]^ To date, only a total of 30 such cases reported in China and other countries.^[5]^ Unusual cases of normotensive PA are identified by hypokalemia or by an incidentally discovered adrenal mass.^[6]^ The mechanisms underlying normal blood pressure in such cases are unknown.

We highlight a case with an atypical presentation of PA with hypokalemia and normal blood pressure, initially expressing the clinical features of anorexia nervosa (AN). The early recognition of PA is crucial to provide appropriate treatment and avoid complications due to PA. Moreover, appropriate and timely management decrease morbidity, mortality, and healthcare expenditure.

## 2 Case report

A 43 years old Chinese woman presented to our emergency department with progressively general weakness for 1 day. She had experienced 1 episode of sudden onset of flaccid paralysis of lower extremities for a couple of minutes, but it spontaneously resolved by itself 5 days before admission. Neither she nor her family members had previously experienced any such attack. She denied any history of vomiting, diarrhea, and abdominal pain. Moreover, there was no history of recent strenuous exercise or the use of diuretics.

On physical examination, her blood pressure was normal (122/71 mm Hg), body temperature was 37°C, respiration rate was 18 breaths per minute and heart rate was 78 beats per minute. She looked very thin with 54 kg body weight and 170 cm body height, indicating low body mass index (18.6 kg/m^2^) (normal range: 18.5–22.9). No flaccid paralysis of the limbs or areflexia of the joints at the time of admission was observed. Other physical examination findings were unremarkable.

The results of biochemical studies on admission were shown in Table 1. The predominant findings were marked hypokalemia (2.2 mmol/L) with metabolic alkalosis (HCO3^–^: 36.8 mmol/L) and renal potassium wasting (transtubular potassium excretion gradient: 9). Moreover, the relatively low urine sodium and chloride levels were also noted. Serum magnesium level, thyroid function test, and cortisol level were within normal limit. Treatment for hypokalemia was initiated by oral and intravenous supplementation of potassium (2.2 mmol/L). Serum potassium level remained low (3.1 mmol/L) after oral potassium supplementation and intravenous infusion of potassium chloride (total potassium supplement, 160 mEq/L within 18 hours).

**Table 1:**
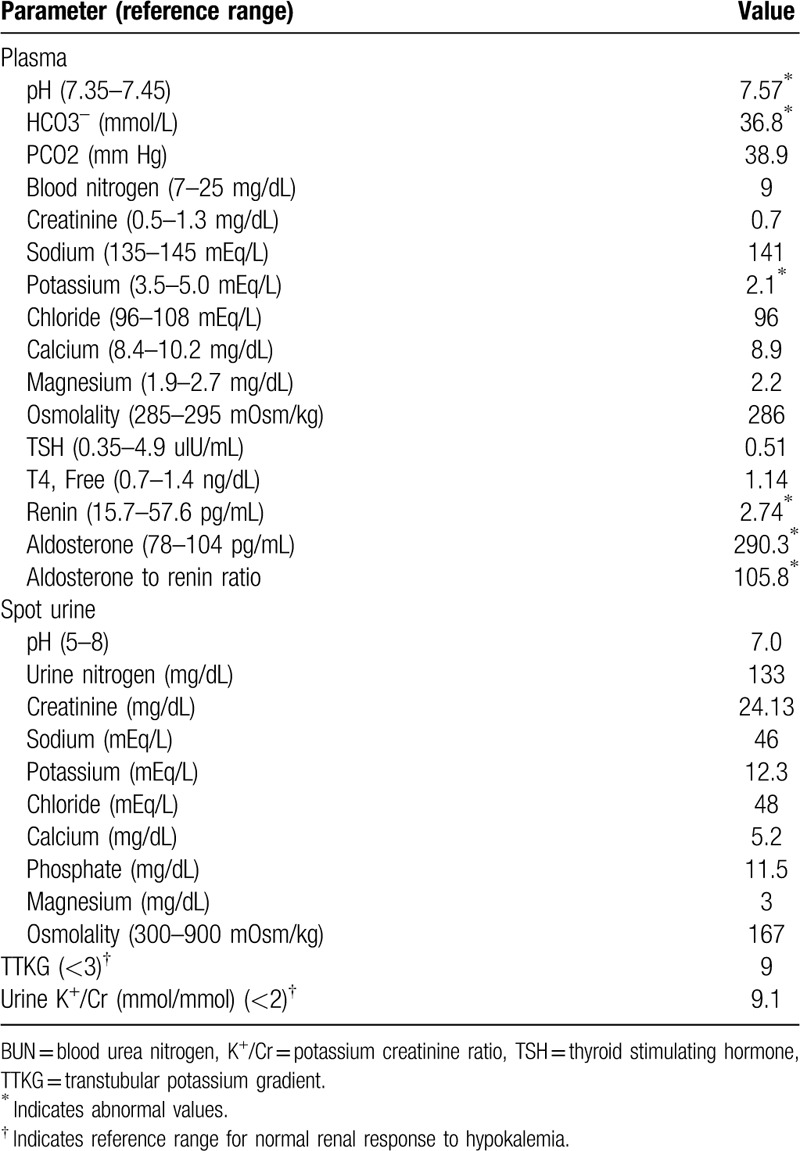
Blood and urine biochemistry at admission.

**Figure 1:**
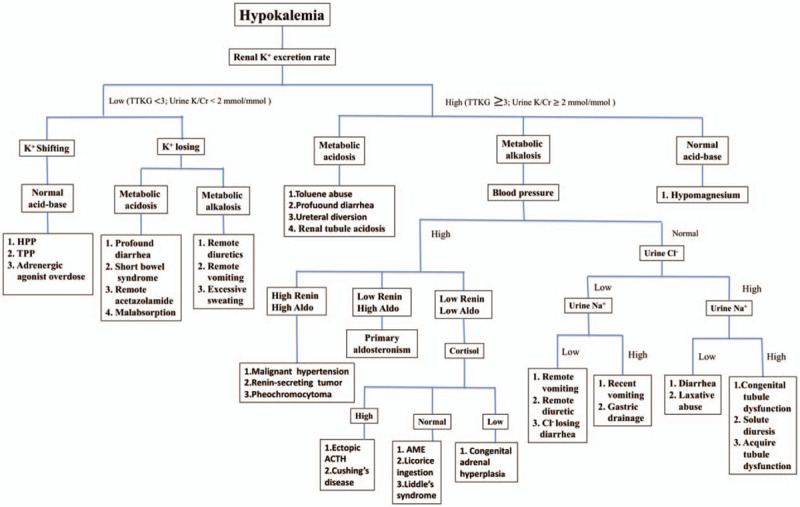
Hypokalemia differential diagnosis flow chart (16–19).

Initially, AN with remote vomiting was highly suspected as the etiology of hypokalemia because laboratory data were compatible with remote vomiting or diarrhea and in accordance with the hypokalemia differential diagnosis flow chart (Fig. 1). She strongly denied the habits of self-induced vomiting or having diarrhea, but she admitted to drinking a lot of water recently to prevent a urinary tract infection.

**Figure 2:**
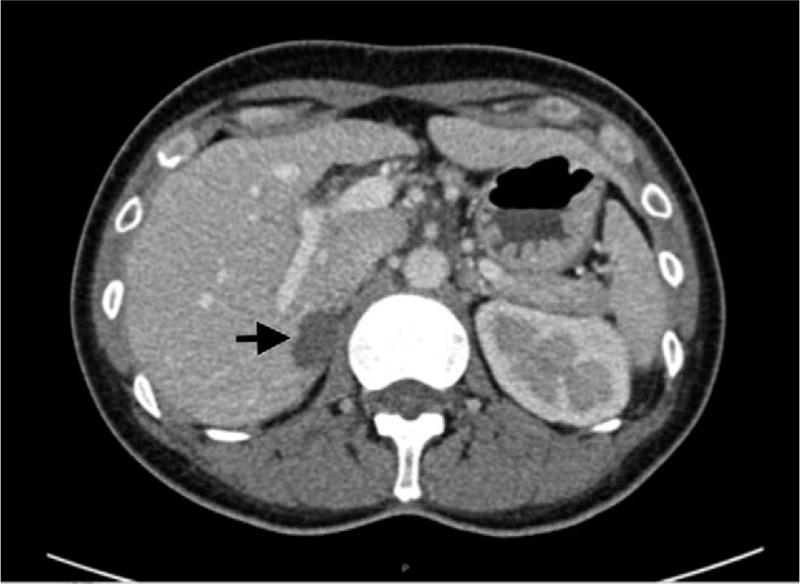
Contrast abdominal CT showing a mass lesion (22 mm) of right adrenal gland (arrow). CT = computed tomography.

Thus, the other etiologies apart from AN were investigated. High aldosterone level (290.3 pg/mL) and low renin level (2.74 pg/mL) with extremely high aldosterone-to-renin ratio (105.8) biochemically indicated the diagnosis of PA. Contrast enhanced computed tomography scan of abdomen revealed a well-circumscribed homogenous mass, 2.2 cm in diameter, in the inner arm of the right adrenal gland, confirming the diagnosis of PA (Fig. 2). Laparoscopic right partial adrenalectomy of right adrenal mass was performed owing to persistent hypokalemia. Macroscopic examination of the extracted adrenal mass found an encapsulated 2.4 × 1.3 cm golden yellow nodule. Microscopic examination concluded to the adenomatous nature of this adrenocortical nodule without malignancy signs. After surgical treatment, biological abnormalities (hypokalemia and metabolic alkalosis) and hormonal status (plasma aldosterone and renin activity) returned to normal level. After discharge, no further hypokalemia was found during a regular one and half year follow-up at the outpatient department without any treatment.

## 3 Discussion

The main clinical manifestation of PA is the combination of hypertension and hypokalemia. Although recent studies have found that the incidence of hypokalemia is only approximately 50% in patients with PA, the vast majority of patients suffer from hypertension.^[7]^ PA results in sodium and fluid retention, leading to extracellular volume expansion. Further, hypervolemia results in hypertension and suppressed renin secretion.^[8]^ Other effects of aldosterone may contribute to increased blood pressure levels in PA. In fact, aldosterone promotes the vascular action of angiotensin II,^[9]^ impairs endothelial function,^[10]^ alters vascular compliance and activates sympathetic ways in central nervous system mechanisms.^[11]^

There have been a few reports of normotensive PA in China and abroad. In 1972, Brooks et al ^[12]^ first reported a normotensive patient with PA. To date, only a total of 30 such cases were reported in China and other countries. Rossi et al^[6]^ analyzed the 26 normotensive patients with PA in the literature, showing that 85% of cases were from Europe and Asia, especially and mainly from Japan. Most of the patients were middle-aged and females. Blood pressure level is the product of cardiac output and vascular resistance. Vascular resistance is the resultant of 2 opposite systems: the vasoconstrictor system which tend to increase blood pressure and the vasodilator system which tend to lower it.^[13]^ Patients with normotensive PA may have lower level of vasoconstrictor system or higher level of vasodilator system. Lower level of vasoconstrictor system may result from lower secretion of, or decreased responses to vasoconstrictor factors, such as angiotensin II and catecholamines. High level of vasodilator system may result from higher secretion of vasoconstrictor factors, such as Prostaglandin E, Kallikreins and nitric oxide, or increased responses to these factors. Excessive urinary kallikrein excretion was found in a patient with normotensive PA.^[14]^

AN, a type of eating disorder characterized by self-enforced starvation and vomiting for the maintenance of body weight, is a psychiatric disease with increased prevalence.^[15]^ Patients with AN usually present with extracellular volume depletion and associated with metabolic alkalosis, hypokalemia, hypophosphatemia, and hyperreninemic hyperaldosteronism.^[16]^ Among these presenting symptoms, hypokalemia is the most commonly encountered and may be due to potassium deficiency from restricting diet, repeating vomiting, and excessive renal loss. In patients with AN, estimated prevalence of hypokalemia was 19% to 20% in studies.^[17]^

Our patient's clinical features presented as AN such as low body mass index and hypokalemia. According to hypokalemia differential diagnosis flow chart^[18–21]^ (Fig. 2), our patient had high renal potassium secretion, metabolic alkalosis, and normotensive blood pressure. Based on the clinical features and laboratory data of our patient, AN with remote vomiting was initially suspected. However, she insistently denied any history of vomiting or induced vomiting, diarrhea, abdominal pain, use of diuretics, and history of recent strenuous exercise. Hypokalemia persisted despite potassium supplementation. Although she had normotensive hypokalemic metabolic alkalosis, PA was later confirmed owing to high plasma aldosterone level contrasting with suppressed plasma renin activity. Lack of hypertension in hypokalemic patient with high renal potassium secretion and metabolic alkalosis should not be exclude PA.

In summary, PA rarely presents with normal blood pressure and hypokalemia, mimicking AN at a young age. We concluded from our case that, for adrenal incidentaloma with hypokalemia, renal potassium wasting, normotension, and metabolic alkalosis, it is necessary to conduct screening tests for the possibility of PA, after the exclusion of other etiologies.

## Author contributions

**Conceptualization:** Yu-Wei Fang, Mei-Lan Tu.

**Supervision:** Ming-Hsien Tsai, Yu-Wei Fang, Mei-Lan Tu.

**Validation:** Ming-Hsien Tsai, Yu-Wei Fang, Mei-Lan Tu.

**Writing – original draft:** Yen-Chu Huang, Mei-Lan Tu.

**Writing – review & editing:** Yen-Chu Huang, Mei-Lan Tu.
